# Character changes and Transcriptomic analysis of a cassava sexual Tetraploid

**DOI:** 10.1186/s12870-021-02963-1

**Published:** 2021-04-19

**Authors:** Xia Chen, Hanggui Lai, Ruimei Li, Yuan Yao, Jiao Liu, Shuai Yuan, Shaoping Fu, Xinwen Hu, Jianchun Guo

**Affiliations:** 1 Agricultural College of Hainan University, Haikou, 571104 China; 2 Key Laboratory of Biology and Genetic Resources of Tropical Crops, Ministry of Agriculture, Institute of Tropical Bioscience and Biotechnology, Chinese Academy of Tropical Agricultural Sciences, Haikou, 571101 China

**Keywords:** *Manihot esculenta* Crantz, Sexual tetraploids, Characters, Transcriptome, Differentially expressed genes

## Abstract

**Background:**

Cassava (*Manihot esculenta* Crantz) is an important food crop known for its high starch content. Polyploid breeding is effective in its genetic improvement, and use of 2*n* gametes in sexual polyploid breeding is one of the potential methods for cassava breeding and improvement. In our study, the cassava sexual tetraploid (ST), which carries numerous valuable traits, was successfully generated by hybridizing 2*n* female gametes SC5 (♀) and 2*n* male gametes SC10 (♂). However, the molecular mechanisms remain unclear. To understand these underlying molecular mechanisms behind the phenotypic alterations and heterosis in ST plants, we investigated the differences in gene expression between polyploids and diploids by determining the transcriptomes of the ST plant and its parents during the tuber root enlargement period. We also compared the characters and transcriptomes of the ST plant with its parents.

**Results:**

The ST plant was superior in plant height, stem diameter, leaf area, petiole length, plant weight, and root weight than the parent plants, except the leaf number, which was lower. The number of starch granules was higher in the roots of ST plants than those in the parent plants after five months (tuber root enlargement period), which could be due to a higher leaf net photosynthetic rate leading to early filling of starch granules. Based on transcriptome analysis, we identified 2934 and 3171 differentially expressed genes (DEGs) in the ST plant as compared to its female and male parents, respectively. Pathway enrichment analyses revealed that flavonoid biosynthesis and glycolysis/gluconeogenesis were significantly enriched in the ST plants, which might contribute to the colors of petiole (purple-red), root epidermis (dark brown), and tuber starch accumulation, respectively.

**Conclusions:**

After sexual polyploidization, the phenotype of ST has changed significantly in comparison to their diploid parents, mainly manifest as enlarged biomass, yield, early starch filling, deep colored petiole and root epidermis. The tetraploid plants were also mature early due to early starch grain filling. Owing to enriched flavonoid biosynthesis and glycolysis/gluconeogenesis, they are possibly resistant to adversity stresses and provide better yield, respectively.

**Supplementary Information:**

The online version contains supplementary material available at 10.1186/s12870-021-02963-1.

## Background

Cassava (*Manihot esculenta* Crantz) is the fourth most widely grown crop in the tropics, one of three major starch crops in the world, and the main food crop in tropical Africa [1]. Its tuberous roots are rich in starch— 20 - 40% of fresh weight and 80% of dry weight, — giving it the highest rank among the known starch crops, and hence cassava is often known as “underground food”, “king of starch”, “special crop” and “energy crop” [2–4]. The breeding studies on this important economic and energy crop are very important and were widely reported. The conventional breeding methods by producing sexual hybrids and selecting natural variation are the common breeding methods used to produce cassava varieties [5]. An excellent cassava variety among the TMS91934 and TMS89/00037–17 cassava clones was identified by observing the leaf morphology [6]. Other breeders have also obtained fine varieties through the intraspecific hybridization of excellent cassava germplasms, such as KU50 and the Rayong series [7]. Although the traditional improvement approach has helped cassava breeding process, it is still far from enough to meet the demand for cassava root yield, starch content, and resistance to abiotic and biotic stresses.

Polyploid breeding has been reported as an effective way in the genetic improvement of crops [8]. The yield of tetraploid *Brassica rapa ssp. (Chinese* cabbage) is 20–30% higher than that of diploid with enhanced resistance to abiotic and biotic stresses [9]. The tetraploid of Hongyang kiwifruit has significantly enhanced disease-resistance to ulcer in vitro than the diploid [10]. In polyploid breeding of cassava, most varieties were autotetraploids that were produced by doubling the number of somatic cell chromosomes by using colchicine to treat axillary buds or stem buds, such as the SC8 and SC124 autotetraploids [11–13]. Another method of colchicine soaking and mixed culture to treat budded stem segments, cotyledon segments, and cluster buds of cassava to obtain SC8 and ARG7 autopolyploids was also used [14].

Breeders now use sexual multiplication mediated by 2*n* gametes to form chromosome recombination [15, 16]. The polyploid offsprings were formed by 2*n* gamete meiosis polyploidization (2*n* gametes were produced by a microspore or megaspore mother cell without meiosis), and the homologous chromosomes of the parents were recombined at a high frequency; thus, the parent alleles can segregate each other into forming offsprings [17–19]. Recombining genes through somatic chromosome-doubling-mitosis polyploidization (somatic cells undergo chromosome doubling during mitosis) is not possible, due to the lack of meiotic recombination. Breeding scientists have successfully developed several excellent varieties of crops through 2*n* gamete polyploidization [15], such as *Primula denticulata* (primrose) [20]*, Vaccinium* section *Cyanococcus* (blueberry) [21], *Populus tomentosa* Carr. (poplar) [22], and *Hevea brasiliensis* (rubber) [23]. In *Solanum tuberosum* (potato) crop, polyploid breeding by 2*n* gametes has helped develop elite varieties (from haploid to hexaploid), which have high protein content [24–26]. Sexual polyploids show desired phenotypic and physiological growth changes in comparison to their diploid parents: the sexual triploid *Tulipa gesneriana* L. (tulip) is commercially important because of its large flowers and bright colors [27], and the sexual tetraploid *B. rapa* ssp. *pekinensis* has high biomass and an increased yield [28]. Sexual polyploids are also more stable than asexual polyploids, because they develop from zygotes so there is no chimera phenomenon [29]. Thus, sexual polyploidization breeding is more valuable in introducing new variation and developing new combinations of alleles in the offsprings.

Despite several reports on sexual polyploidization in other plants, study on 2*n* gamete polyploidization in cassava has rarely been reported. Our research group has successfully obtained sexual tetraploid (ST) plants of cassava by hybridizing 2*n* female and male gametes obtained by treating the cassava megaspore and microspore inflorescence by colchicine and dimethyl sulfoxide (DMSO) to form 2*n* gametes at sexual stage [30, 31]. Reports show that phenotypic alterations in polyploids could be related to changes in the expression of particular groups of genes after chromosome doubling [32–34]. These gene expression changes have been found in many natural and synthesized polyploid plants [35], such as, a changed expression of both new genes and gene groups in allopolyploid/−autotetraploids *Arabidopsis* [33]. The importance of RNA-mediated gene regulation in polyploidization of *Brassica napus* [36, 37], significant changes in the genes related to flavonoid biosynthesis and lipid in seed development after polyploidization in *Gossypium* [38, 39], and the haphazard loss and silencing of genes after polyploid formation in natural or synthetic allotetraploids of *Tragopogon miscellus* [40] are some examples of changed gene expression. But the changes in gene expression and regulation networks in cassava polyploids need further study.

Development of the next generation high-throughput sequencing technology is a revolutionary breakthrough in genomics and transcriptional detection. By measuring transcriptome-wide gene expression, we can analyze the differences in gene expression between polyploids and diploids, which can help understand the mechanism behind polyploid phenotypic variation. Transcriptome sequencing techniques have been used in many artificial and naturally occurring polyploid plants, and it has been demonstrated that there is a significant difference in gene expression between polyploids and diploids [35, 41–43]. These DEGs were usually associated with plant hormone synthesis, signal transduction, transcription factors, and glucose metabolism, etc., which were associated with phenotypic differences in growth and reproduction between tetraploids and diploids [34, 44, 45]. The underlying molecular mechanisms of the phenotypic alterations and heterosis in ST plants of cassava are still unknown and need further study. To address this concern, we investigated the differences in gene expression between polyploids and diploids by determining the transcriptomes of the ST plant and its parents during the period of enlargement of tuberous roots. We also compared the characters and transcriptomes of the ST plant with its parents. Our study will help reveal the underlying molecular mechanisms of phenotypic variations in ST plants at the functional genome level. It will also pave the way for further research on the structure and function of key controlling genes that are related to important agronomic traits, such as root formation, root yield, and adversity stresses in the sexual polyploids of cassava.

## Results

### Polyploidy identification of the hybrid cassava variety

**Fig. 1:**
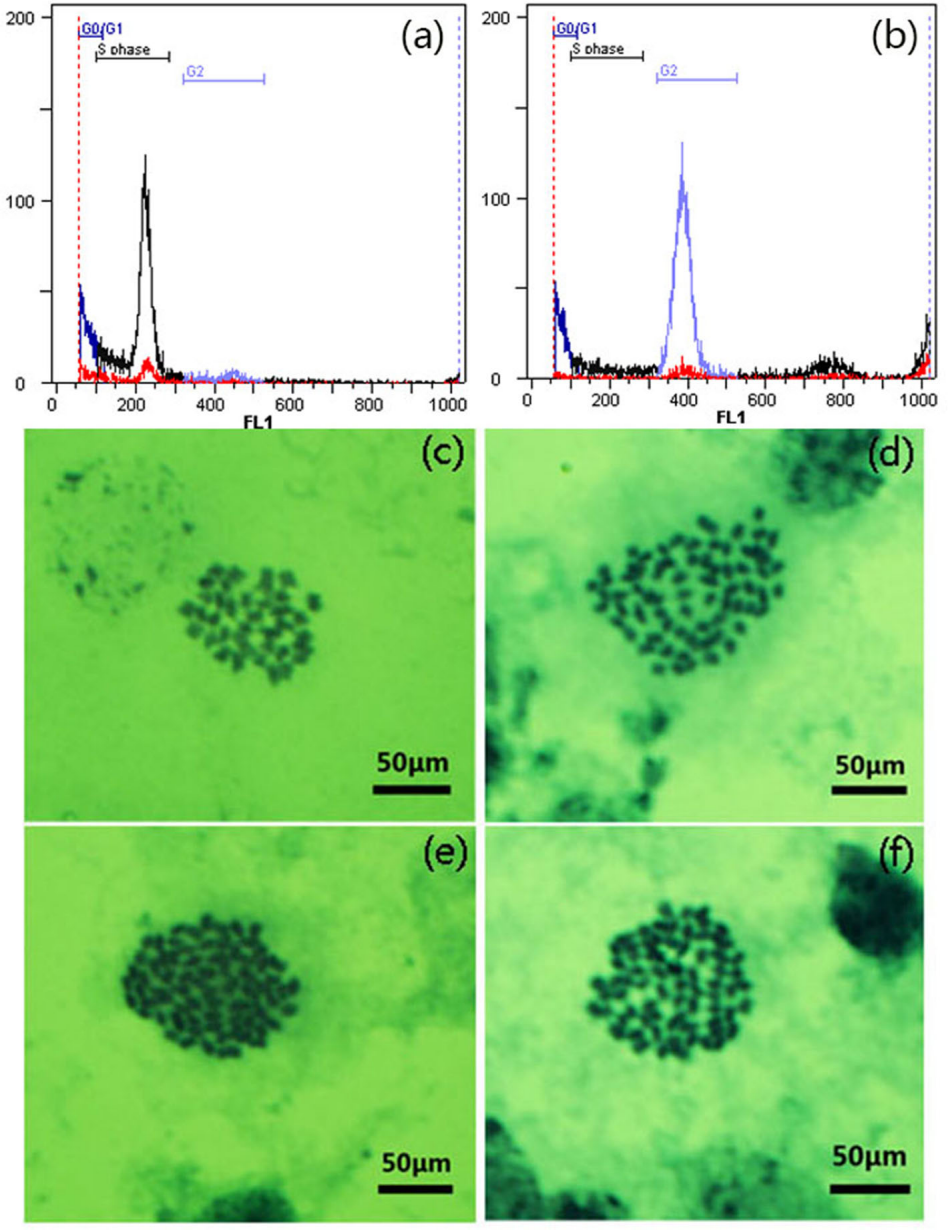
Ploidy determination of the hybrid cassava variety. **a** Diploid cassava variety images captured by flow cytometry; **b** Hybrid cassava variety images captured by flow cytometry; **c** Chromosomes of diploid foliage, 2*n* = 2*x* = 36; **d** Chromosomes of hybrid variety F1 foliage, 2*n* = 2*x* = 72; **e** Chromosomes of clones F2 foliage, 2*n* = 2*x* = 72; **f** Chromosomes of clones F3 foliage, 2*n* = 2*x* = 72

The hybrid cassava seedlings were derived from the sexual cross of the parents SC5(♀) × SC10(♂). The ploidy of the hybrid cassava seedlings (F1) was confirmed by flow cytometry and leaf cell chromosome counts. The DNA content of the diploid parent shows a main peak at channel 200 (Fig. 1a), and the hybrid cassava plants show twice the DNA content of the control diploids, with the main peak at channel 400 (Fig. 1b). Their chromosome number is twice that of the diploid parent (Figs. 1c and d). We did not find chimeric diploid cells, which confirmed that the hybrid cassava variety is a uniform tetraploid (Fig. 1). The ploidy stability of the clone lines (F2/F3) was confirmed by counting the leaf cell chromosomes. The results show that the ploidy of cassava sexual tetraploids (ST) is uniform and stable (Fig. 1e, f).

### Characters of the cassava ST plants in the field

**Fig. 2:**
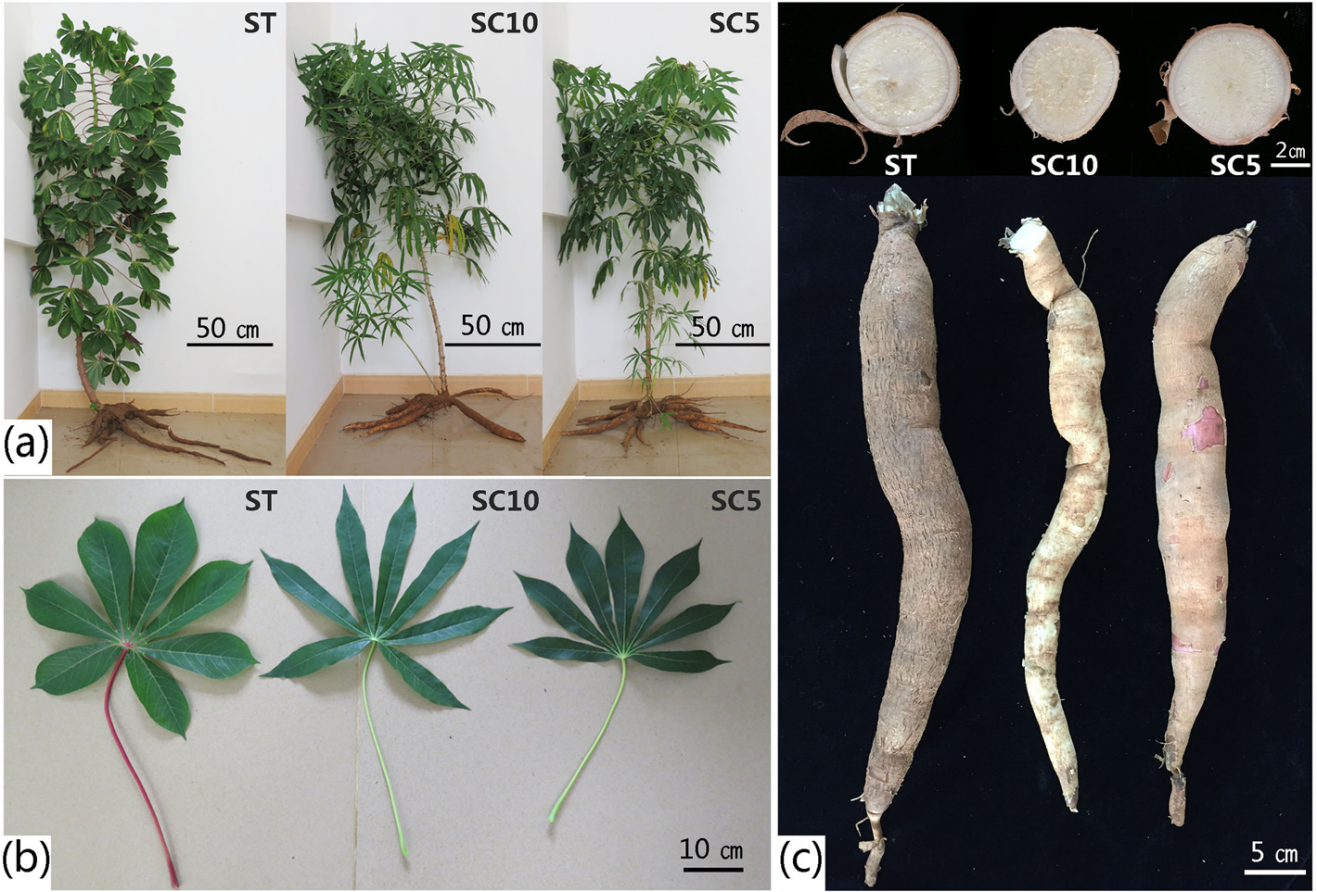
Phenotypic observation of the cassava sexual tetraploids and their diploid parents. **a** Sexual tetraploid and its parent diploid plants five months after planting; **b** Leaves of the cassava sexually tetraploid and its diploid parents; **c** Tuber roots of the cassava sexual tetraploid and its diploid parents (the top of the pictures are the cross-sections of the tuber roots of ST, SC10, and SC5, respectively)

The results showed that the ST plants had large leaves with a rounder leaf shape, thicker veins, and a longer and purple-red petiole as compared to the parent plants (Fig. 2b). The root epidermis of the ST plant was darker (Fig. 2c) and its phloem was thicker than that of its parent plants (Fig. 2c). The average values of the above ground parts of the ST plants including plant height, stem diameter, leaf area, petiole length, and plant weight were higher than those of the parent plants, except the leaf numbers, which were lower (Table 1). For the underground parts, the root weight of ST plants was higher than that of the parent plants, but there was no significant difference in the length or diameter of the roots (Table 1). The leaf net photosynthetic rate was higher in the ST plant as compared to its parent plants, while root dry matter rate does not show any significant difference (Table 1).

**Table 1:** Phenotypic characters of the cassava sexual tetraploid (ST) plants and their parent plants in the field

Parameter	Sexual Tetraploid (ST) Plants	SC10(♂) Plants	SC5(♀) Plants
Height (cm)	205.00 ± 9.57A	195.00 ± 8.66A	167.67 ± 16.62B
Stem diameter (cm)	4.51 ± 0.65A	2.65 ± 0.33B	3.13 ± 0.32A
Leaf area (cm^2^)	518.19 ± 23.57A	308.33 ± 35.66B	306.16 ± 59.37B
Leaf number	78.67 ± 10.26A	107.00 ± 25.24AB	129.30 ± 6.11B
Petiole length (cm)	36.33 ± 3.51A	21.83 ± 5.92B	18.50 ± 2.31B
Aboveground part weight (kg)	4.10 ± 1.22A	1.53 ± 0.31B	1.49 ± 0.07B
Underground part weight (kg)	4.08 ± 1.73A	2.33 ± 0.33B	2.69 ± 0.17B
Length of root (cm)	39.55 ± 21.18A	36.70 ± 12.16A	40.26 ± 15.16A
Diameter of root (cm)	3.97 ± 0.80A	3.05 ± 0.81B	3.42 ± 0.98A
Net photosynthetic rate (μmol·m^2^·s^− 1^)	11.87 ± 1.23A	6.05 ± 1.94B	8.51 ± 1.46B
Root dry matter rate (%)	28.51 ± 0.52A	28.44 ± 0.16A	32.56 ± 1.03A

Note: Different capital letters represent significant difference at 0.05 level (Duncan, *P* = 0.05)

**Fig. 3:**
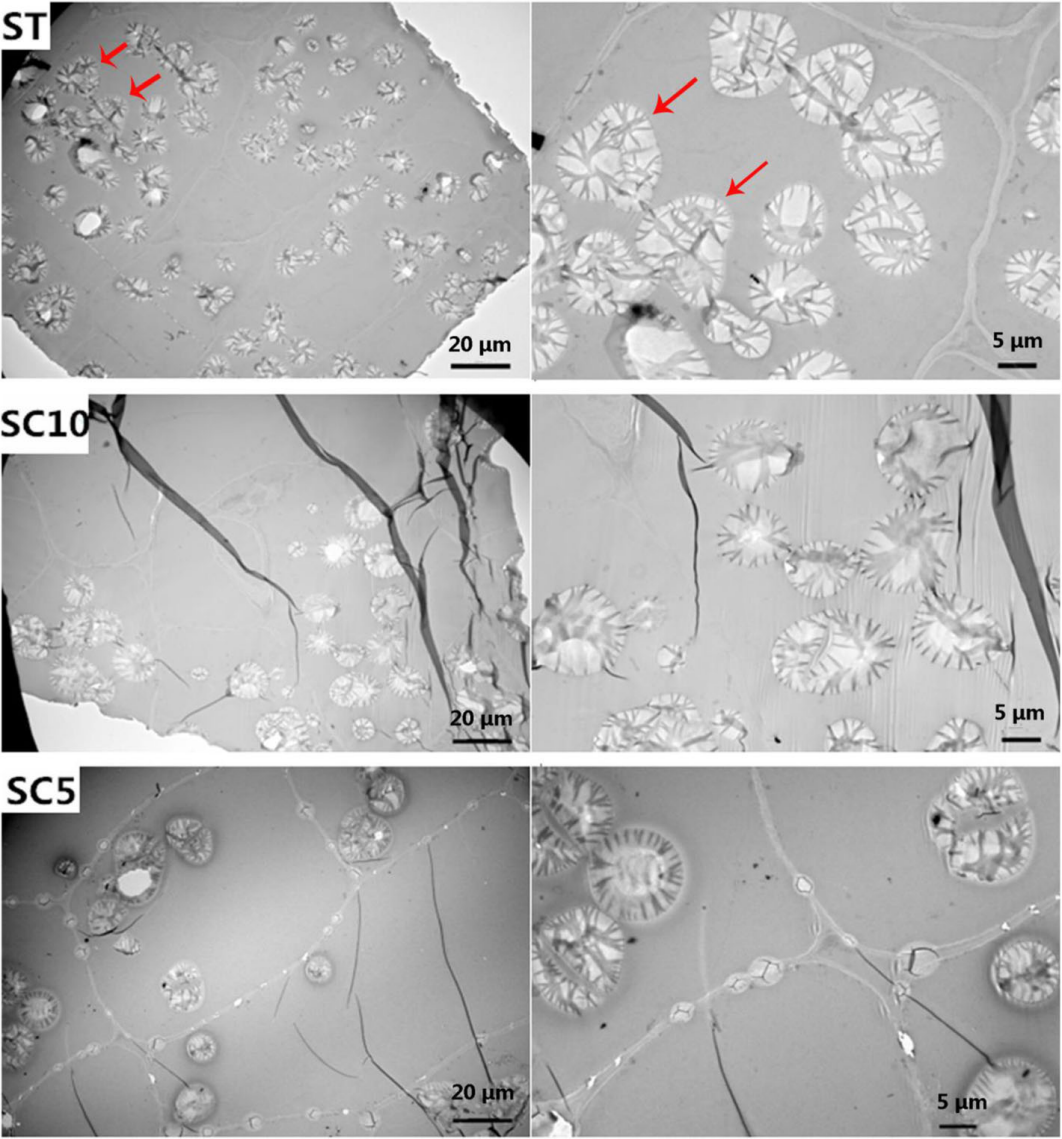
Transmission electron microscopy of starch granules in the tuber roots of the ST, SC10, and SC5, respectively

The starch granules in the tuber roots of the ST, SC5 and SC10 were investigated five months after planting by transmission electron microscopy of their cross-sections. The number of starch granules was higher in the roots of ST plants than that in the parent plants after five months (tuber root enlargement period) (Fig. 3), but there was no difference after ten months (tuber root maturity period). Additional file (Fig. S1) shows details of this observation. Our results indicate that the starch grains in ST plants filled in earlier than in the parents. We did not observe any significant difference in cell size between ST plants and parent plants in these two periods (Figs. 3 and S1). In the ST plant, two or three starch grains stuck together to form irregularly shaped amyloplasts, while in its parents, most of the amyloplasts had one starch granule, while few of them had two or three starch granules (the red arrow in Fig. 3). The density of the “zebra stripes” was also different in the starch granules of ST plants as compared to that in parent plants. Our findings indicate changes in the internal structure of the starch granules in ST plants.

### Transcriptome data acquisition

**Fig. 4:**
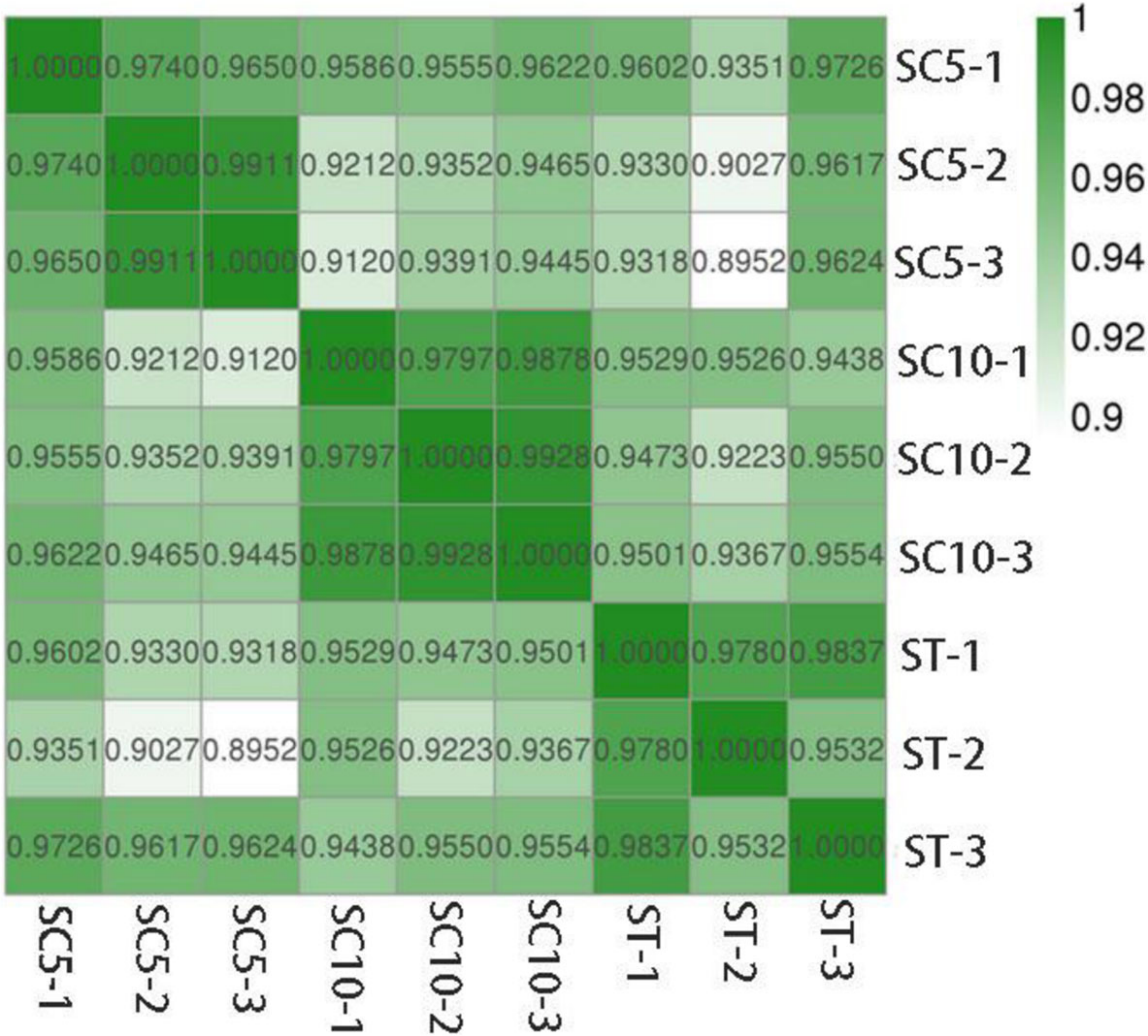
The correlation heat maps among the three bioreplications for each variety. The abscissa and ordinate represent each sample, and each block of abscissa and ordinate represent the correlation between X and Y samples. The darkest (green) color in the graph represents the largest Pearson correlation coefficient between the X and Y samples and the lightest (white) color represents the smallest Pearson correlation coefficient between the X and Y samples

In total reads, the proportion of unmapped reads was approximately 20%, the proportion of unique mapped reads was approximately 79.5%, and the proportion of multiple mapped reads was approximately 0.5% (Table 2). The correlation coefficient heatmap analysis and reproducibility assay showed that the three replicates of each variety were highly correlated (Pearson correlation > 0.95) and repeatable (Fig. 4). An additional movie file (Fig. S2) shows this observation in more detail. The results demonstrate that the duplicate data are valid for gene expression analysis.

**Table 2:** Statistical comparison of the obtained reads (after mapping to ribosomes and cassava reference genome)

Sample		Total Reads	Unmapped Reads	Unique Mapped Reads	Multiple Mapped Reads	Mapping Ratio
Sexual tetraploid	ST-1	45,643,992	9,383,481 (20.56%)	36,039,967 (78.96%)	220,544 (0.48%)	79.44%
	ST-2	43,351,046	8,404,178 (19.39%)	34,723,052 (80.10%)	223,816 (0.52%)	80.61%
	ST-3	45,864,544	8,783,710 (19.15%)	36,845,820 (80.34%)	235,014 (0.51%)	80.85%
Pollen parent	SC10–1	32,017,874	6,403,495 (20.00%)	25,466,457 (79.54%)	147,922 (0.46%)	80.00%
	SC10–2	39,603,108	7,917,056 (19.99%)	31,476,866 (79.48%)	209,186 (0.53%)	80.01%
	SC10–3	34,141,878	6,714,779 (19.67%)	27,256,093 (79.83%)	171,006 (0.50%)	80.33%
Female parent	SC5–1	38,473,060	7,372,557 (19.16%)	30,894,599 (80.30%)	205,904 (0.54%)	80.84%
	SC5–2	33,531,478	6,647,600 (19.82%)	26,672,456 (79.54%)	211,422 (0.63%)	80.18%
	SC5–3	38,578,920	7,598,678 (19.70%)	30,759,608 (79.73%)	220,634 (0.57%)	80.30%

***Note:*** Unmapped reads are the reads that cannot be mapped to the reference genome and their ratio of total reads. Unique mapped reads are the reads that are uniquely mapped to the reference genome and their ratio of total reads. Multiple mapped reads are the reads that are mapped to multiple locations of the reference genome and their ratio of total reads

**Fig. 5:**
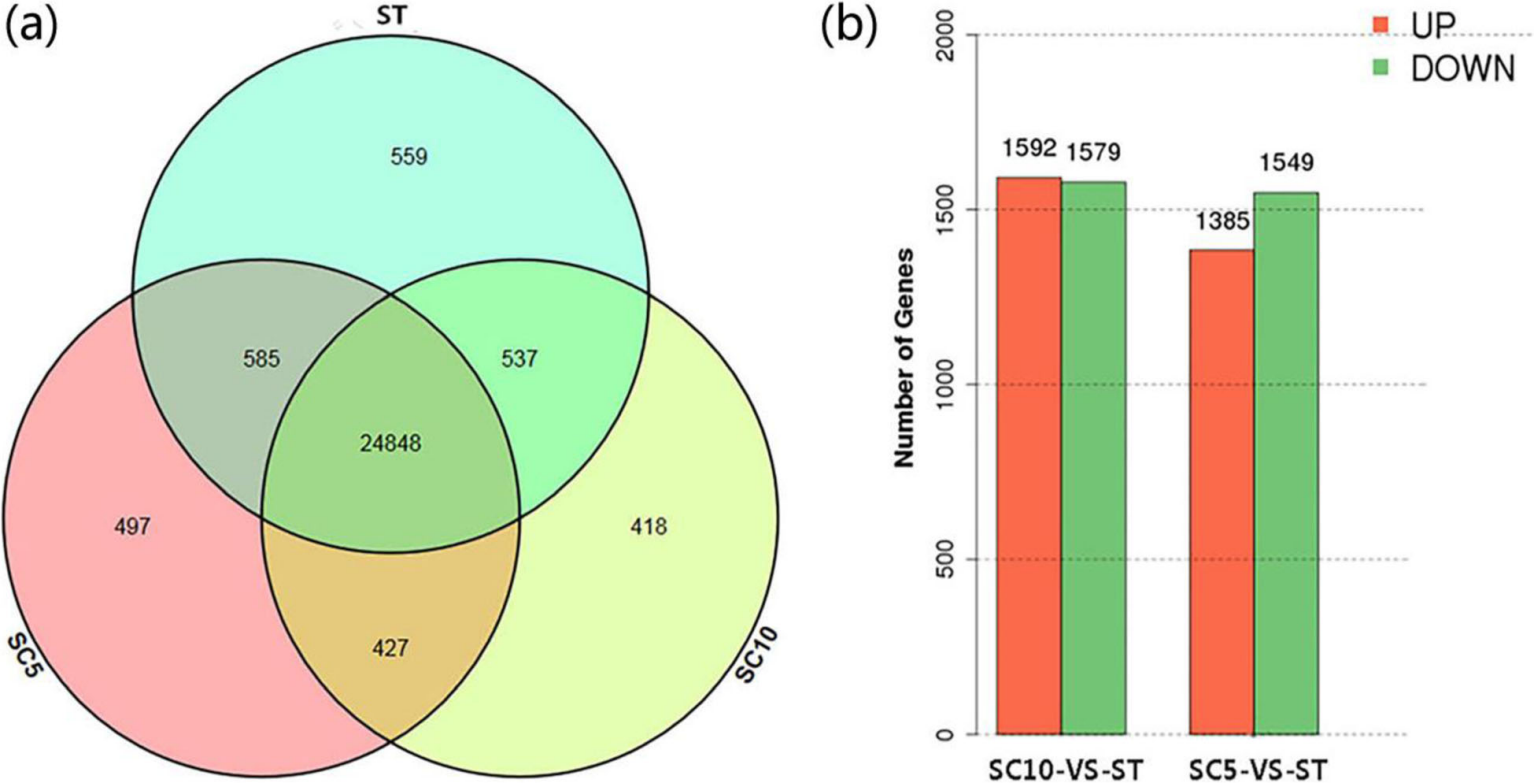
**a** Venn diagrams for transcriptome analysis of the expressed genes detected in the three cassava varieties; **b** The histograms of differentially expressed gene between samples. Ascissa: pairs of samples; ordinate: number of differentially expressed genes; red represents up-regulated genes; green represents down-regulated genes

In total, of 27,274 known genes that make up 82.54% of the entire reference genome, we detected 611 potential new genes. Most of these genes were expressed in all three plant materials, and some genes were exclusively expressed in specific ones such as SC5 (497), SC10 (418), and ST (559) (Fig. 5a).

### Analysis of differentially expressed genes between the ST plants and its parent plants

We identified 2934 and 3171 DEGs in the ST plant as compared to its female and male parents, respectively (Fig. 5b). Our results show that only a small number of genes were differentially expressed among all the detected genes in ST plants and parent plants, indicating that gene expression had no obvious genome-wide change after polyploidization.

### Gene ontology functional and KEGG pathway enrichment analyses of DEGs

**Fig. 6:**
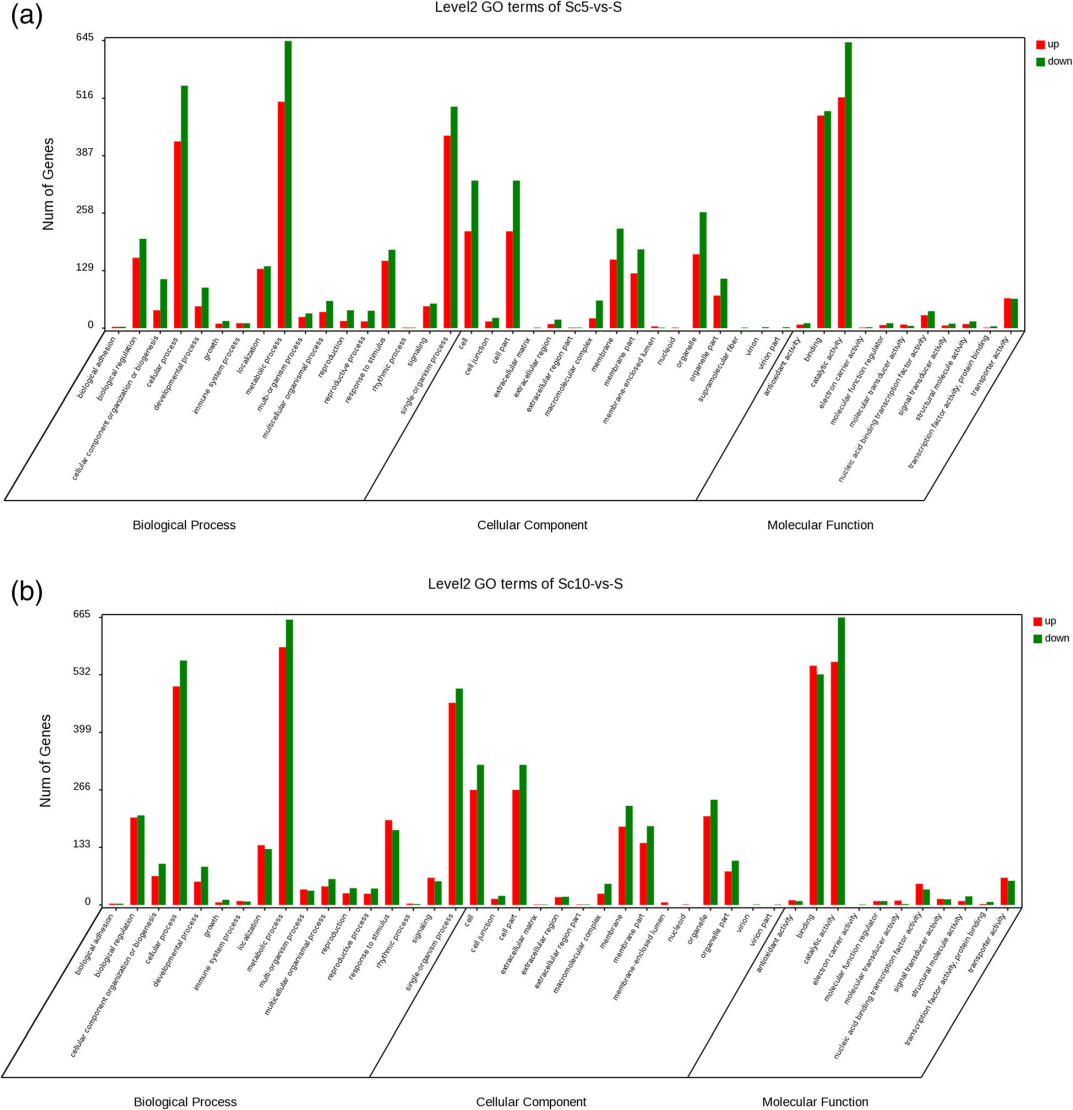
**a** GO classification of DEGs between the ST and SC5; **b** GO classification of DEGs between the ST and SC10

The GO function enrichment analysis showed that the DEGs between the ST and its parents were grouped into 44/43 GO terms, respectively, which were included in cellular processes, cellular components, and molecular function (Figs. 6a and b). Compared to the female parent, the DEGs were mostly enriched in the biological process of cellular processes, metabolic processes, single-organism processes, biological regulation, response to stimulus, and localization. The enrichment distribution of ST plants as compared to the male parent was very similar to the female parent, except that no DEGs were enriched in the biological process of supramolecular fiber. The DEGs were enriched in the process of flavonoid biosynthesis and metabolism as compared to the female parent.

**Fig. 7:**
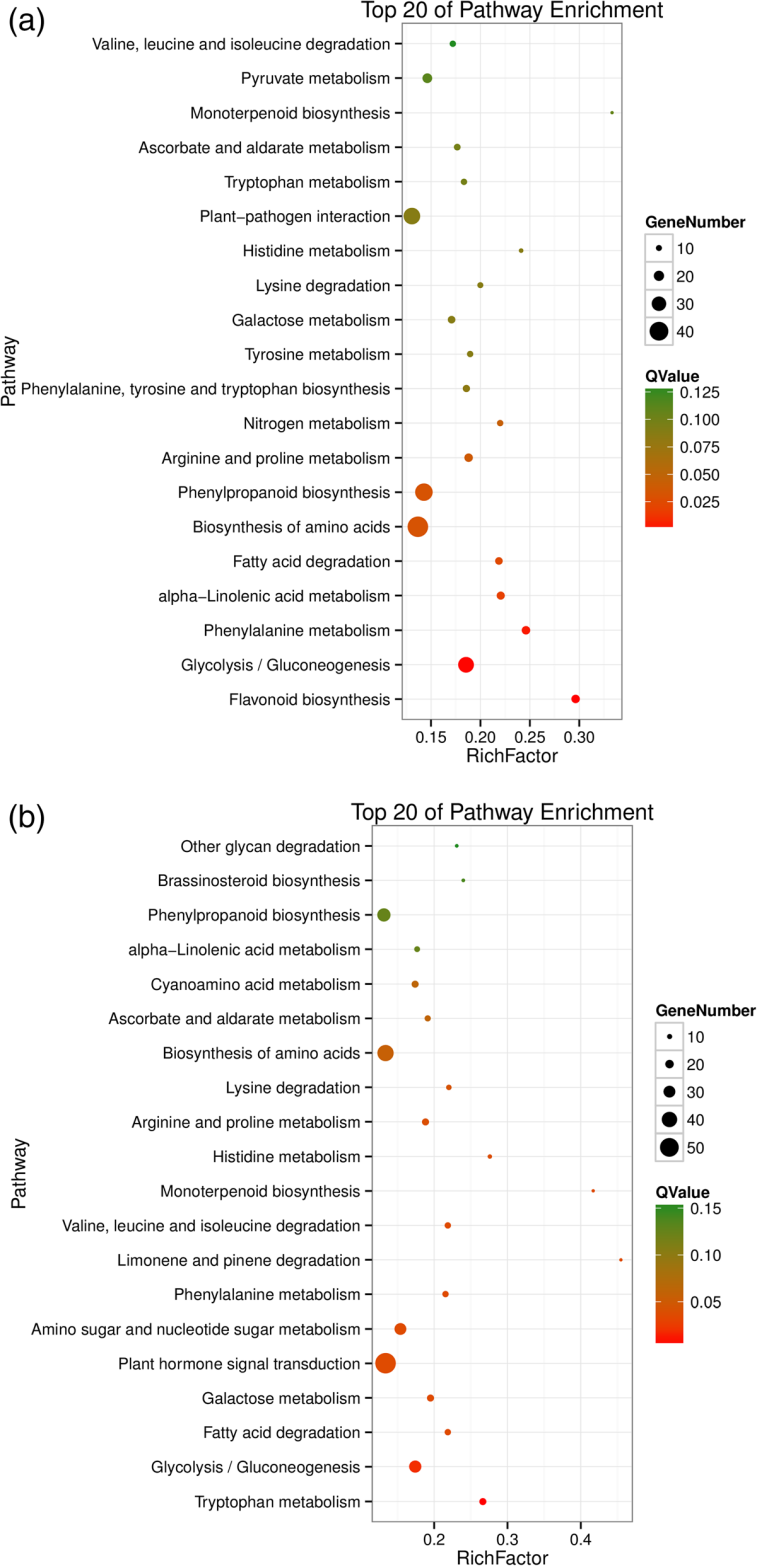
**a** The top 20 of the KEGG pathway enrichment of DEGs between the ST and SC5; **b** The top 20 of KEGG pathway enrichment of DEGs between the ST and SC10

**Fig. 8:**
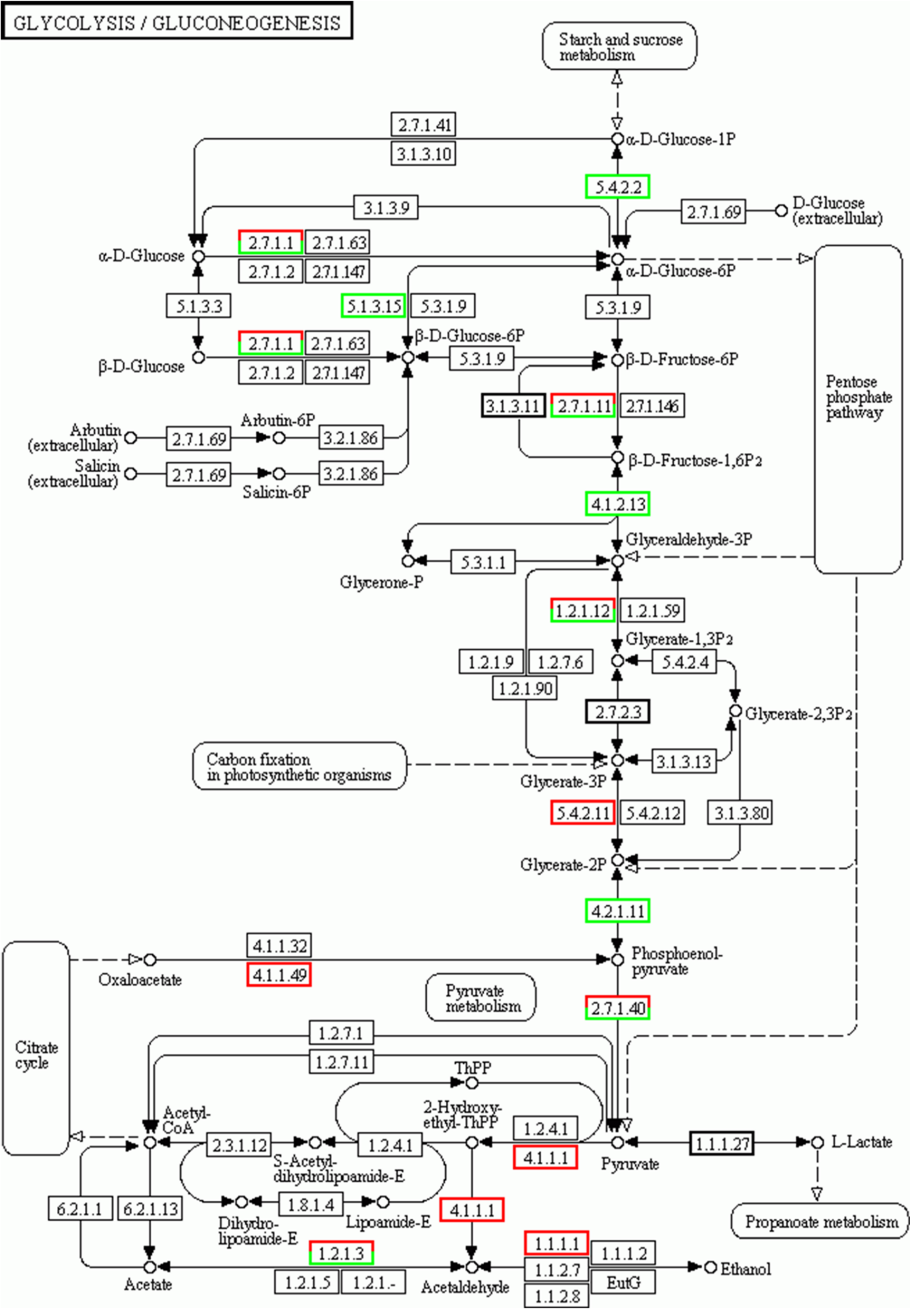
Glycolysis/gluconeogenesis pathway of the cassava roots; the positions of the up-regulated DEGs are marked in red, and the positions of the down-regulated DEGs are marked in green

Figures 7a and b show the top 20 enrichment pathways, which are similar to the GO enrichment results. Compared to the female parent SC5, the DEGs were most enriched in flavonoid biosynthesis (Fig. 7a). Many DEGs were secondarily enriched in the process of glycolysis/gluconeogenesis in both female and male parents (Figs. 7a and b). Glycolysis/gluconeogenesis were closely related to starch accumulation in cassava roots (Fig. 8), which reveals that sexual tetraploidy may affect the starch accumulation.

### Verification of DEGs involved in glycometabolism by quantitative real-time RT-PCR (qRT-PCR)

**Fig. 9:**
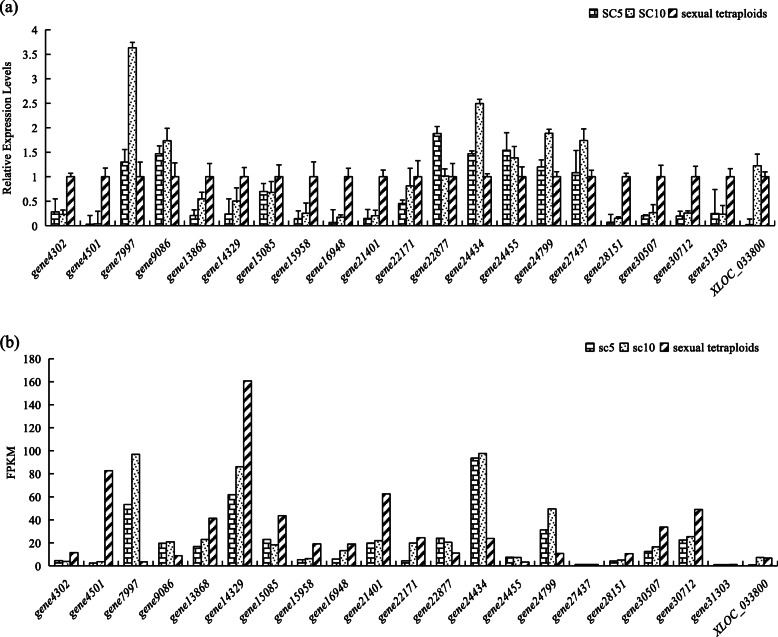
**a** Relative expression levels of DEGs determined by q-PCR; **b** Transcriptome data for DEGs expression

To verify the accuracy of the RNA-seq sequencing results, 21 DEGs involved in glycolysis/gluconeogenesis were selected for qRT-PCR. Figure 8 illustrates the up-regulated DEGs between the ST and its parent plants in red boxes and the down-regulated DEGs in green boxes. As in the transcriptome sequencing analysis, the RNAs extracted from tuberous roots of three cassava plant materials during the tuber enlargement period were used for qRT-PCR (which were the same as used in the transcriptome sequencing analysis). The relative expression change trends of qRT-PCR are in accordance with that of RNA-seq (Fig. 9), which demonstrates that the transcriptome comparison data are reliable.

## Discussion

The sexual polyploid of 2*n* gametes is the most important polyploidy in plants, as germ cells are more sensitive to the environment than somatic cells [46]. In the present study, we obtained artificial ST plants by artificially inducing 2*n* gametes in female and male parents and hybridizing them [47]. We studied the characters of ST plants, and observed that they showed improved plant characters as compared to its parent plants. The leaves of ST plants were larger in size as compared to the parent plants: the leaf area was significantly higher, the petiole was longer, and the harvested organ roots in the expansion stage were also Significantly higher than diploid parents. We also observed a decrease in leaf number in ST plants, indicating the polyploid nature where plants are without branches. This trait can make cassava easily manageable in the field and is more conducive to dense planting of cassava. These findings show that the growth characters of cassava ST plants are consistent with that of general polyploids. Compared with asexual cassava polyploid, since the ST of cassava is hybridized by 2*n* gamete pathway [47] and then directly developed through zygote, there is no chimerism. It will not degenerate and return to diploidy in the process of reproduction. Its ploidy homozygosity and asexual reproduction stability have been verified in this study. It is worth mentioning that we did not observe flowering and fruiting in three generations of cassava ST clones, hence ST plant cannot be used as a hybrid parent to further obtain sexual triploid or other hybrids. Usually, after chromosome replication, the chromosomes in the cell nucleus show meiotic pairing disorder, resulting in reduced growth rate and infertility of the offspring, which is a common phenomenon in polyploid species [48]. Transmission electron micrographs showed many starch grains in ST plants in the root enlargement period (five months after planting), while the roots of the two parent plants almost had not even started to fill (Fig. 3). The early filling of starch grains might be associated with a higher leaf net photosynthetic rate. This is an important finding in selection of early-maturing cassava varieties.

Generally, the polyploid genome behavior impacts heterosis, and allopolyploids (both natural and synthetic) often exhibit “genomic superiority” [49, 50]. The phenotypic and physiological changes of polyploids result from the changes in gene expression after gene duplication to generate changed gene regulatory pathways [31, 36, 51–53]. In our study, we observed that some of these DEGs were involved in biological regulation, metabolic processes, localization, response to stimulus, and developmental processes. Our findings are in sync with that of the autotetraploid of *Morus alba* L. (mulberry) [42]. In *Paulownia fortunei* polyploidization, gene expression variation caused epigenetic-related changes [35, 43], and in *Betula platyphylla* (birch), the biosynthesis and signal transduction of indoleacetate (IAA) and ethylene were altered after genome duplication, which contributed to phenotypic changes [44]. Our results indicated that the number of genes in cassava did not change significantly after sexual tetraploidization, but the gene expression changed, and the phenotype and physiological and biochemical changes in cassava STs might be regulated by these DEGs.

Our study on pathway enrichment analysis showed that two metabolic pathways flavonoid biosynthesis and glycolysis/gluconeogenesis showed significant differences in enrichment. The petiole and tuber root epidermis of ST plants were purple-red and dark brown, respectively, which were markedly different from those of their parents. The significantly enriched metabolic pathway of “flavonoid biosynthesis” might result in the changed color of the petioles and the root epidermis in ST plants due to accumulation of anthocyanins (flavonoids). The flavonoids also act as antioxidants in plant stress resistance: *Arabidopsis* showed oxidative and drought tolerances by overaccumulation of antioxidant flavonoids [54], the antibacterial activity of *Zingiber officinale* Rosc. (ginger) had a correlation with flavonoid content [55], postharvest physiological deterioration (PPD) of cassava roots was negatively correlated with phenolics and carotenoids and positively correlated with anthocyanins, flavonoids, and melatonin [56, 57]. The roots of ST plant were significantly enriched in “flavonoid metabolic pathways”. In a separate study we observed that its root tuber had stronger resistance to PPD than its parents. The photosynthetic rate, the ability to synthesize sucrose in cassava leaves, and the ability to utilize sucrose in cassava tuber roots play key roles in the process of starch accumulation in cassava [58–61]. The ST plant has a higher net photosynthetic rate than the parent plants and is significantly enriched in the metabolic pathway of glycolysis/gluconeogenesis that might affect its yield and slightly change the dry matter composition of its roots.

## Conclusions

The present study investigated the characters and genome-wide gene expression profiling of cassava sexual tetraploid and its diploid parents, with an aim to illustrate the molecular mechanisms of character changes after sexual polyploidization. Overall, the phenotype and physiological characters of cassava sexual tetraploids are different as compared their diploid parent plants, including greater biomass, higher photosynthetic rate, purple color of the petioles and the root epidermis and some other beneficial traits. The higher net photosynthetic rate and significantly enriched in glycolysis/ gluconeogenesis” metabolic pathways in cassava sexual tetraploids as compared to its parent plants might be associated with the early filling starch grains, increased yield, and dry matter composition of its roots. The significantly enriched flavonoid biosynthesis metabolic pathway might result in the changed color of the petioles and the root epidermis in the sexual tetraploids. Further research on genes responsible for economically important agronomic traits in the sexual polyploids of cassava can help produce better varieties on a large scale, leading to bulk production of cassava starch not only for food purpose but also for non-food purposes such as in paper and pharmaceutical industries.

## Methods

### Plant materials

The experimental parent plant materials, *Manihot esculenta* Crantz South China No.5 (SC5) and 10 (SC10) were obtained from Institute of Tropical Crops Resources Research, the Chinese Academy of Tropical Agricultural Sciences. The cassava sexual tetraploid (ST) plants were obtained from hybridization between 2*n* female (SC5) and 2*n* male (SC10) gametes by the following methods:

1. **2*n*** **gamete induction:** Based on the correlation between the meiosis of cassava megaspore/ microspore mother cells and the morphological characteristics of inflorescence and pistil/ stamen, the pistil with 2.8–3.5 mm length/ stamen and 1.0–1.7 mm diameter, with most megaspore/ microspore mother cells in meiosis prophaseIwere selected to induce 2*n* male (SC10) and female (SC5) gametes using 0.3% (w/v) colchicine and 1% (v/v) DMSO by dipping cotton.
2. **Hybridization:** In the flowering stage of cassava, 2*n* female gametes (SC5) were used for interspecific hybridization with 2*n* pollens (SC10).
3. **Uniform tetraploid selection:** Several hybrid mature seeds were collected, and sown to produce plants (F1); F1 were then propagated vegetatively to produce F2 clones, which in turn produced F3 clones. Identification of their ploidy and homozygosity was done by measuring DNA content and counting chromosome number from leaves of F1, F2, and F3 clones. The uniform tetraploid clones with stable phenotypes were used in the following experiments.

Three cassava plant materials (SC5, SC10, and ST) were propagated vegetatively in the Hainan University Agricultural College, Danzhou campus, Haikou, China. All biological replicates were selected randomly from different clones.

### Polyploidy identification in hybrid plants

The ploidy and homozygosity of F1, F2 and F3 plants were identified by the following methods. The DNA content of leaves from the hybrid plant F1 and its diploid parent were evaluated by flow cytometry. A total of 0.5 g young leaves collected from near the top of the cassava plants were, placed in a Petri dish with lysis buffer (45 mM MgCI_2_, 30 mM sodium citrate, 20 mM MOPS, 0.1% v/v Triton X-100), then chopped with a blade, treated for 2–3 min, filtered with a 300 mesh filter to collect the filtrate, then centrifuged for 5 min at room temperature at 1500 rpm. The precipitate was dissolved in 1 mL of sheath fluid and centrifuged for 5 min at room temperature and 1500 rpm. Finally, the precipitate was mixed with 5 mL DAPI dye and stained for 0.5–2 h in dark conditions. The polyploid detection was performed using FACSC alibur Flow Cytometry (BD, USA), and data were analyzed using CellQuest Pro software. The diploid parent plants were used as controls.

The leaf chromosomal samples from the hybrid plants F1, F2, and F3 and their diploid parents were prepared by an enzymatic desorption method [62]. The bronze cassava leaves were collected between 9 am and 12 am, pretreated with p-dichlorobenzene saturated liquid at room temperature for 1–2 h, then rinsed 4–5 times with tap water and 3–5 times with distilled water. The leaves were then fixed in anhydrous ethanol and glacial acetic acid (3: 1) for 10–24 h. The leaves were rinsed with distilled water 3–5 times for 5 min each time, then kept in distilled water for 30 min for prehypotonic infiltration. The enzyme hydrolysis of the leaves was performed with a mixture of enzymes (0.75% cellulase and 3.75% pectinase) at 37 °C for 2–4.5 h (the volume ratio of the leaves to enzyme was approximately 1:4). After gently and carefully washing the leaves with distilled water 2–3 times, they were then kept in distilled water for 30 min for low permeability; and were finally fixed for 15 min in the (anhydrous ethanol and glacial acetic acid as already discussed. The leaf was carefully put on a glass slide free from bubbles and was quickly crushed with a tweezer. The large pieces of residue were clipped out and air dried. Then, it was stained with 10% (w/v) Giemsa staining solution (pH 7.0) for 20 min. The excess dye was washed off with water, and the leaf was air dried. The cells in metaphase division were selected for chromosome number calculation under the microscope.

### Phenotyping

Phenotypes of the ST and its parent diploid plants were investigated in 5-month-old plants. The ST, SC5, and SC10 plants cultivated in a field for five months were selected for measuring of plant height, stem diameter, leaf area, petiole length, leaf number, leaf net photosynthetic rate, aboveground and underground plant weight, length and diameter of roots, and root dry matter rate. The fourth completely expanded leaf was selected for leaf measurements (leaf area, petiole length, leaf number, and leaf net photosynthetic rate), and the leaf area was calculated by using the scanning method and ImageJ software. The net photosynthetic rate of the cassava leaves was detected by using a GFS-3000 Photosynthesis Tester (WALZ, Germany Products) [63]. Three biological samples each from ST, SC5, and SC 10 plants were randomly selected from three independent plants for each experimental analysis. Each experiment was carried out in triplicates.

The starch granules and amyloplasts in the tuberous roots of 5-month-old ST, SC5, and SC10 plants were observed under transmission electron microscopy [64]. Three samples each from ST, SC5, and SC 10 plants were randomly selected from three independent plants for each experimental analysis.

### RNA extraction and transcriptome sequencing

Total RNA was extracted from fresh tuberous roots of 5-month-old ST, SC5, and SC10 plants by using an Ominiplant RNA Kit (DNase1) (ComWin, Beijing, China) following the manufacturer’s instructions. Three samples each from ST, SC5, and SC 10 plants were randomly selected from three independent plants for each experimental analysis. Each experiment was carried out in triplicates. The total mRNA was enriched by oligo (dT), then fragmented using fragmentation buffer and reversely transcribed into cDNA by using a NEB#7530 Kit (NEB#7530 Kit, New England Biolabs). The cDNA fragments were end repaired, poly(A) added, and ligated to Illumina sequencing adapters, then the products were purified with the AMPure XP Beads (1.0X) Kit following the manufacturer’s instructions. Transcriptome sequencing was performed using Illumina Hi Seq TM2500 (Gene Denovo Biotechnology Co., Guangzhou, China).

### Alignment with the reference genome and transcript reconstruction

The original image data obtained through this sequencing were converted into raw data by base calling. To ensure the quality of data, the original data were performed before informatic analysis, and the redundant data were discarded through data filtering. After strictly filtering these clean reads, including the adapter-containing reads, more than 10% N reads, the low-quality reads (the bases with quality values Q ≤ 20 that accounted for more than 50% of the entire read), and finally, the high-quality clean reads were used for subsequent informatic analysis.

To further prevent ribosome contamination, a short-reads comparison tool Bowtie2 [65] was used to align high-quality clean reads with the ribosome database. After removing the reads belonging to ribosomes, the remaining data of each sample were aligned to the cassava reference genome by using the transcriptome data alignment software TopHat2 (v.2.0.3.12) for transcriptome assembly and further analysis. The alignment parameters were as follows: maximum read mismatch was 2; the distance between mate-pair reads was 50 bp; the error of the distance between mate-pair reads was ±80 bp [66, 67].

After aligning with the reference genome, the unmapped reads (or those poorly mapped) were re-aligned with Bowtie2. The enriched unmapped reads were split into smaller segments that were then used to search for potential splice sites. The sections and the positions of these short segments were predicted as well. A set of splice sites were built with initial unmapped reads by Top Hat2 without relying on the known gene annotation [68].

The reconstruction of the transcripts was carried out with Cufflinks, together with Top Hat2, to identify new genes and new splice variants of the known ones. The reference annotation-based transcript (RABT) algorithm was preferred. Cufflinks constructed faux reads according to the reference to make up for the influence of low coverage sequencing. All the reassembled fragments were aligned with the reference genes and the similar fragments were removed. Different replicas of each group were merged into a comprehensive set of transcripts by using Cuffmerge. The comprehensive transcripts from multiple groups were used for further downstream differential expression analysis.

### GO functional and KEGG pathway enrichment analysis of differentially expressed genes (DEGs)

To compare the differentially expressed genes (DEGs) in the ST and parent plants, the expression value of each gene was calculated using the fragments per kilobase of transcript per million mapped reads (FPKM) method. This method can eliminate the effect of differences in gene length and sequencing on gene expression, and the results can be directly used to compare gene expression differences between different samples. We used false discovery rate (FDR) (the *P* value after FDR corrected) and log2 (FPKM_ST_/FPKM_diploid_) to screen for differential genes at FDR < 0.05 and |log2FC| > 1.

To further understand the function of DEGs between the ST and its parent plants, gene ontology (GO) and Kyoto encyclopedia of genes and genomes (KEGG) databases were used for functional enrichment classification of these genes. All DEGs were mapped to GO terms in the Gene Ontology database (http://www.geneontology.org/) to calculate the numbers of genes for each term, and significantly enriched GO terms in the DEGs compared to the genome background were defined by a hypergeometric test. The *P* value [69] is calculated as follows:

$$ P=1-\sum \limits_{i=0}^{m-1}\frac{\left(\begin{array}{l}M\\ {}\kern0.5em i\end{array}\right)\left(\begin{array}{l}N-M\\ {}\kern1em n-i\end{array}\right)}{\begin{array}{l}N\\ {}n\end{array}} $$

where, *N* is the number of all genes with GO annotation, *n* is the number of DEGs in *N*, *M* is the number of all genes that were annotated to certain GO terms, and *m* is the number of DEGs in *M*.

Pathway enrichment analysis was identified by the Kyoto encyclopedia of genes and genomes (KEGG) database. The *P* value [63] is calculated in the same manner as in the GO term analysis. In this formula, *N* is the number of all genes with KEGG annotation, *n* is the number of DEGs in *N*, *M* is the number of all genes annotated to specific pathways, and *m* is the number of DEGs in *M*.

The raw data of the cassava transcriptome used here have been deposited in the SRA database of the NCBI (accession number SRP151951).

### Real-time quantitative RT-PCR (q-PCR) verification

Total RNA was extracted from tuberous roots of three cassava plant materials (SC5, SC10, and ST) using RNAgen Plus plant total RNA extraction reagent (Tiangen Biochemical Technology Co., Ltd., Beijing, China) according to manufacturer’s instructions, and then reverse transcription was performed. The relative mRNA expression of the DEGs was analyzed by qRT-PCR using a set of gene-specific primers (Table 3) that were designed based on BLAST analysis of the cassava genome database (http://www.phytozome.net/cassava). The reverse transcription and qRT-PCR reaction system and specific operation steps refer to our published paper [70]. Three technical replicates for each sample were analyzed.

**Table 3:** Primers used in qRT-PCR verification

Gene ID	Forward Primer (5′ to 3′)	Reverse Primer (3′ to 5′)
*Gene 4501*	ACACCGCAGGACAAGTCATTCGT	TGCCACCTCTACTTCTTCAATCACA
*Gene 7997*	CAGCCTTCCGCCTCCATTATC	GCAAGACGTTTCCCGCAGGTA
*Gene 21,401*	TGTCCAGCACTACGGGTCTTGTT	GAAACAATGGAGTCTGGCCCTTG
*Gene 24,434*	CTCTCTCCATCAAAGCTTCATCTTCT	CCTTCTTTCGAAGACCACTAGTTCCT
*Gene 4302*	TTGCTACCTTCTTCCACTTCTCATC	CTTCACCGAGACATTCCCAAACT
*Gene 9086*	TTATTTACCAGTAGCAAGGCTGTCTT	CCAGCACTTGAGGTCTTCTTCAGAT
*Gene 15,958*	AAGGCTGCACTGATTGTTGTATTC	GGGCTAGCCAAGATAGGGTAAAC
*Gene 24,455*	CATGGCTAAGCACAGGAGTGAGA	AGCTGGTTGTACTTGGCAAGACG
*Gene 24,799*	ACGGGCTAACAGTTCCAGGAGAC	GCCACAAGCTAATGCAGGACCAA
*Gene 27,437*	ATCGAGATCTCTCTCTTCCATTTTC	TAACTTTAATATCACGGTGCTTCCA
*Gene 28,151*	GAAATGAACGGTGAACCAGAGG	ACAGCTTTTCGACCATAGGCAC
*Gene 30,507*	GGAGAGTGTAGGAGAGGATGTAGATG	CAGTTTTGAACAAAGGTTGCTCTTC
*Gene 31,303*	CTGAAGCAGAGAAAGAGAGGAAAGA	AAATAATGGATTCTGACCCTTAGCC
*Gene 16,948*	ATCTCGTACAAAGGGAGGACCAATT	AGCGTACCCTGCATTTAGCTCGTTA
*Gene 13,868*	TTACAGCTCCTTGTAACCCATC	ACATCTGTCCAATCCTTTACTCGC
*Gene 14,329*	TCCTGTCTCCTGCCTTTCCTCTA	TCATACTGTCCACAACACGAGTAAC
*Gene 22,171*	CAACAGGATGGCTTGTCAACACT	CCTTCAAGAGGCTACCGGAGTGT
*Gene 22,877*	GACTGAGAATCACAGGGCACTAAA	AAGAGCAGAAGCAGAAGCAGAAGT
*Gene 30,712*	ATCTCGTACAAAGGGAGGACCAATT	CCGAGGTAGAAGAGTCTGAGGATTAG
*XLOC_033800*	TTACAGCTCCTTGTAACCCATC	CTTGGGTCTTAGCTTCCTCTTT
*Gene15085*	TCCTGTCTCCTGCCTTTCCTCTA	GACCGAGAGTGGATTCATGAGAG

## Supplementary Information

**Additional file 1.**

## Data Availability

The datasets supporting the conclusions of this article are available in the SRA database of NCBI repository (accession number: SRP151951).

## Abbreviations

SC5: *Manihot esculenta* Crantz South China No.5
SC10: *Manihot esculenta* Crantz South China No.10
ST: Cassava sexual tetraploid
DMSO: Dimethyl sulfoxide
DEGs: Differentially expressed genes
