# Supplementary material for: Character changes and Transcriptomic analysis of a cassava sexual Tetraploid
Source: BMC Plant Biol. 2021 Apr 19;21:188. doi: 10.1186/s12870-021-02963-1 (PMC8056498; doi:10.1186/s12870-021-02963-1)
Supplement: Supplementary file 1 — Additional file 1. [file 12870_2021_2963_MOESM1_ESM.doc]

­­
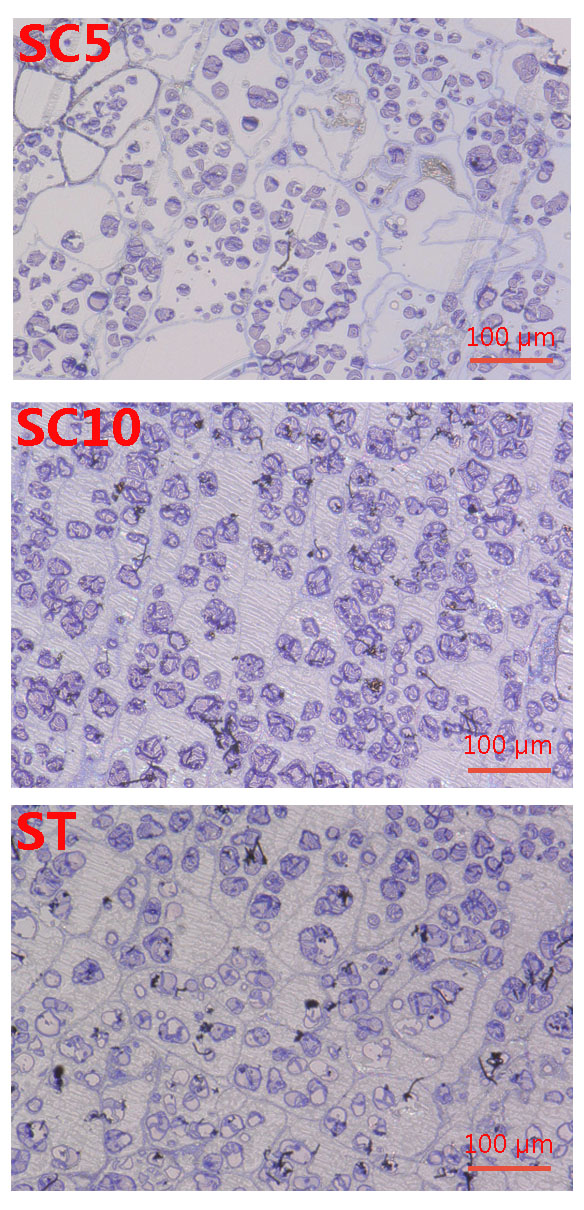


**Figure S1** Semi-thin slice of root starchy granules of ST, SC10, and SC5 in the tuber root mature period.


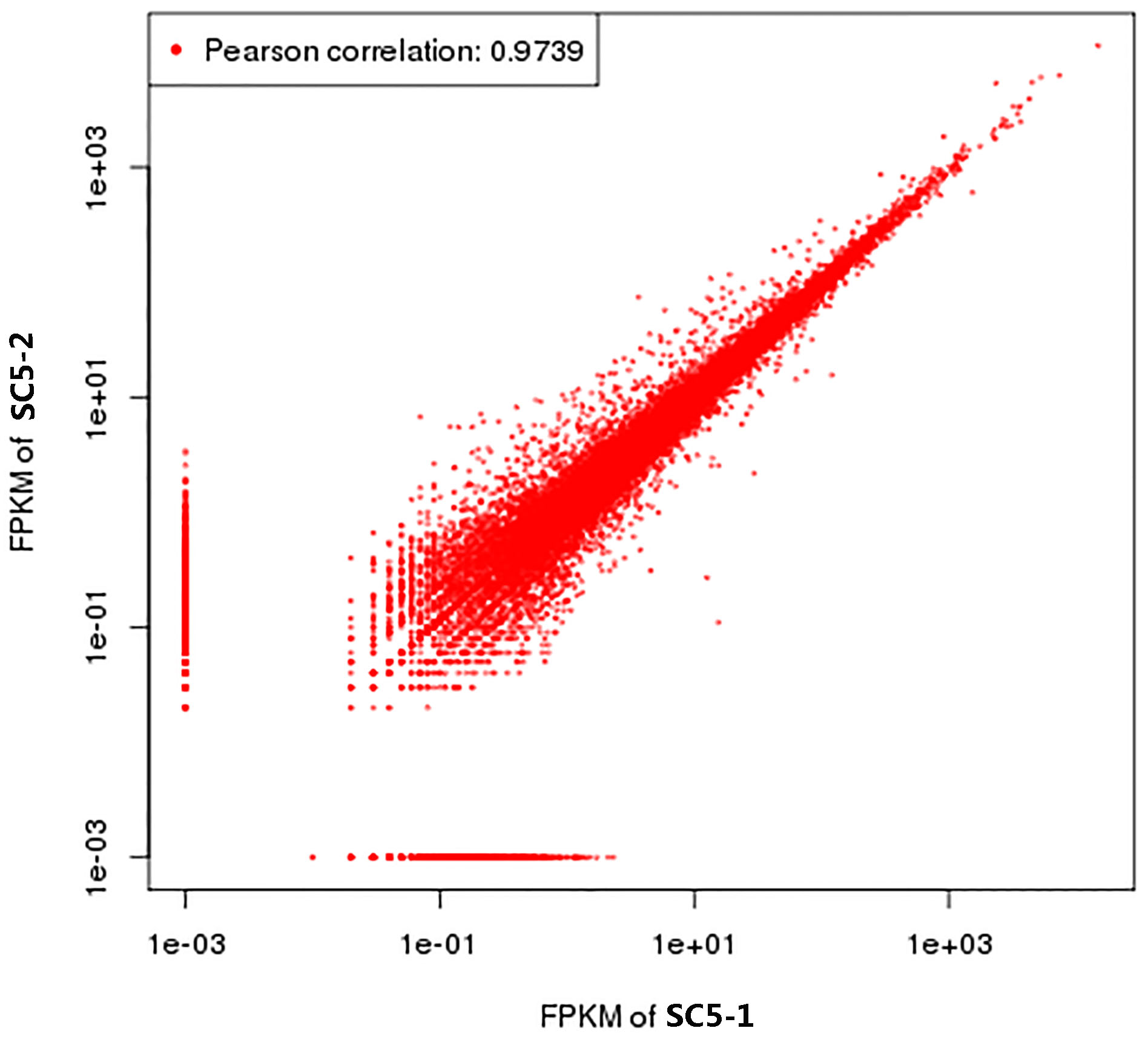

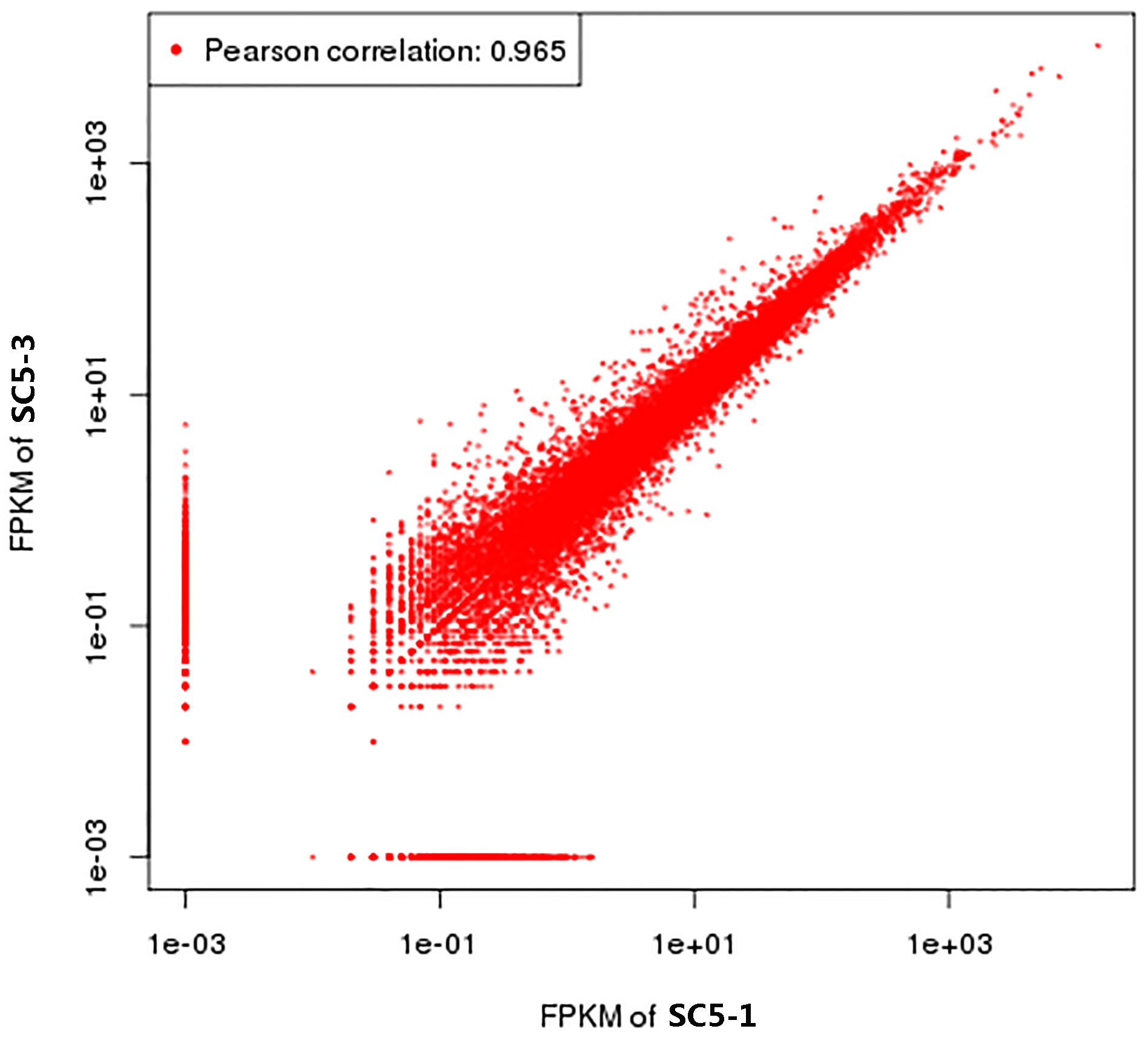

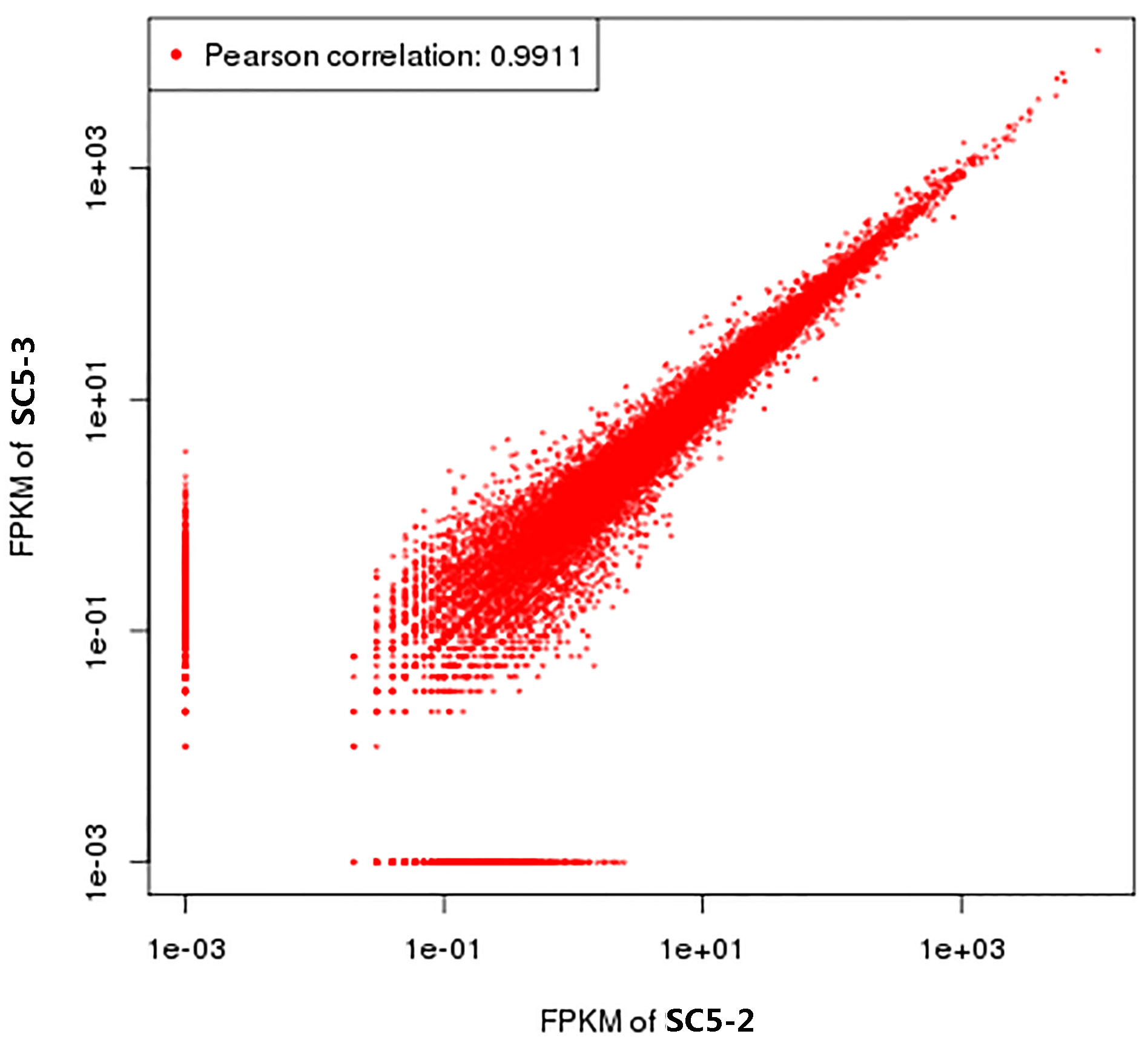

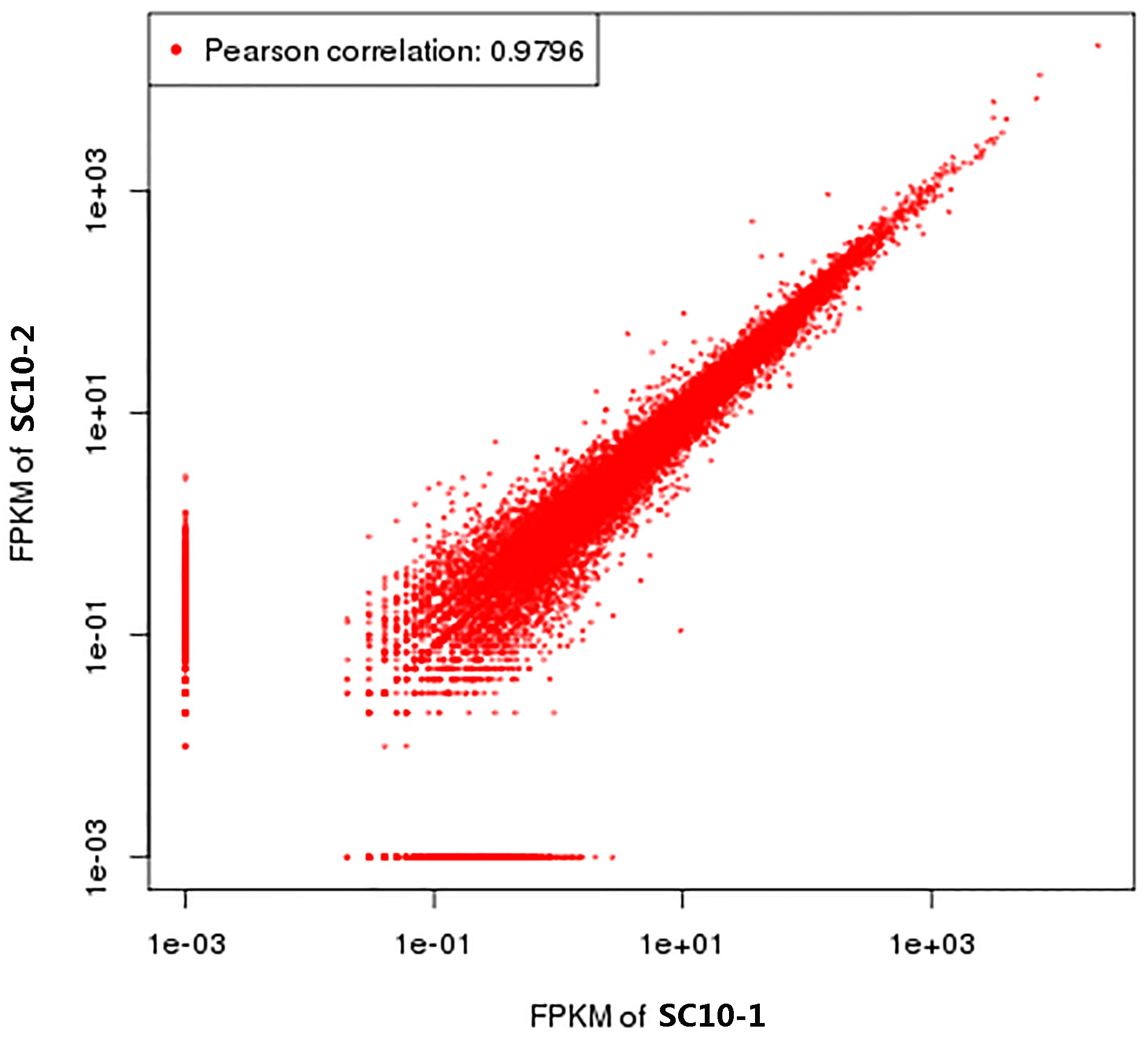

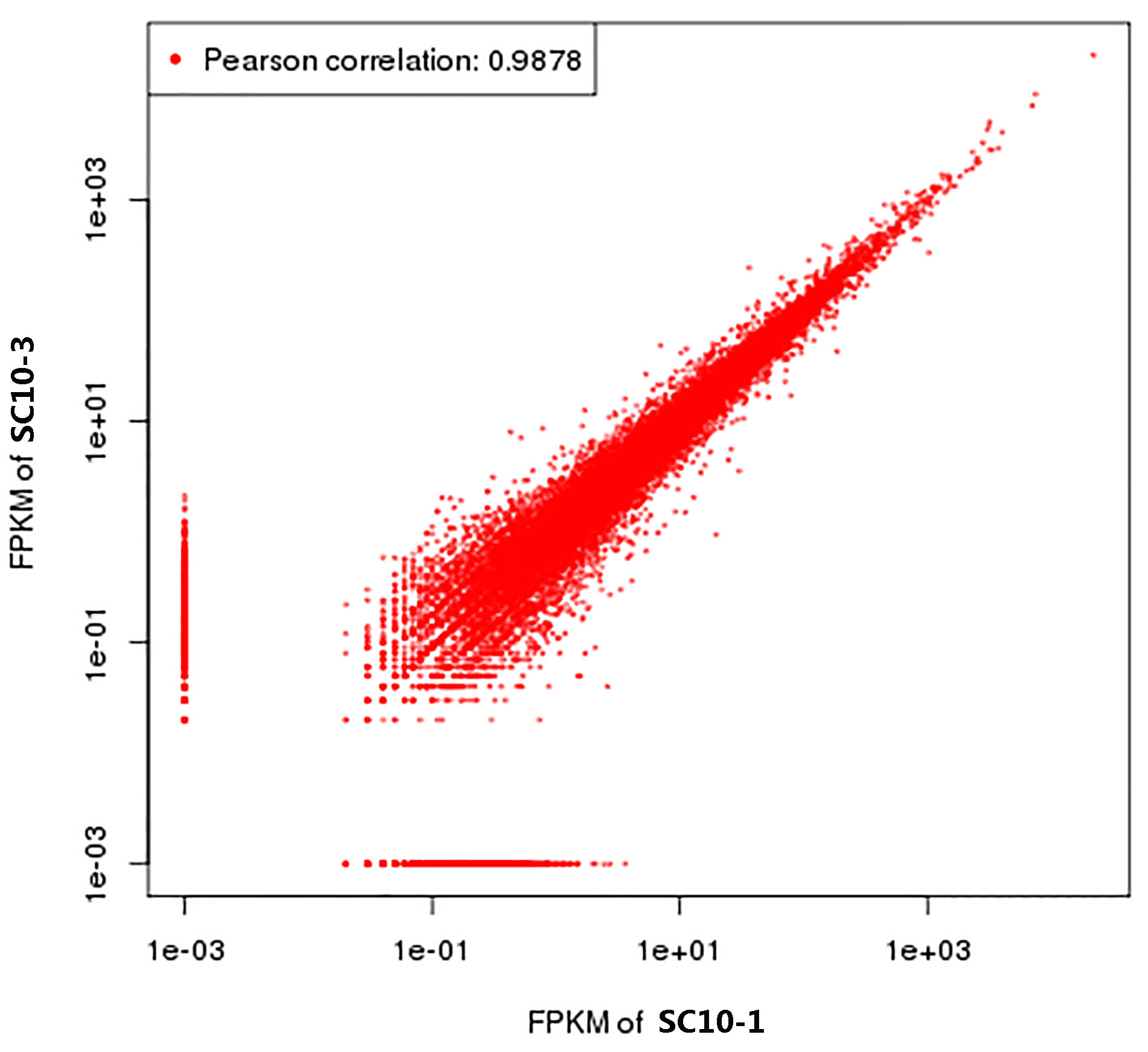

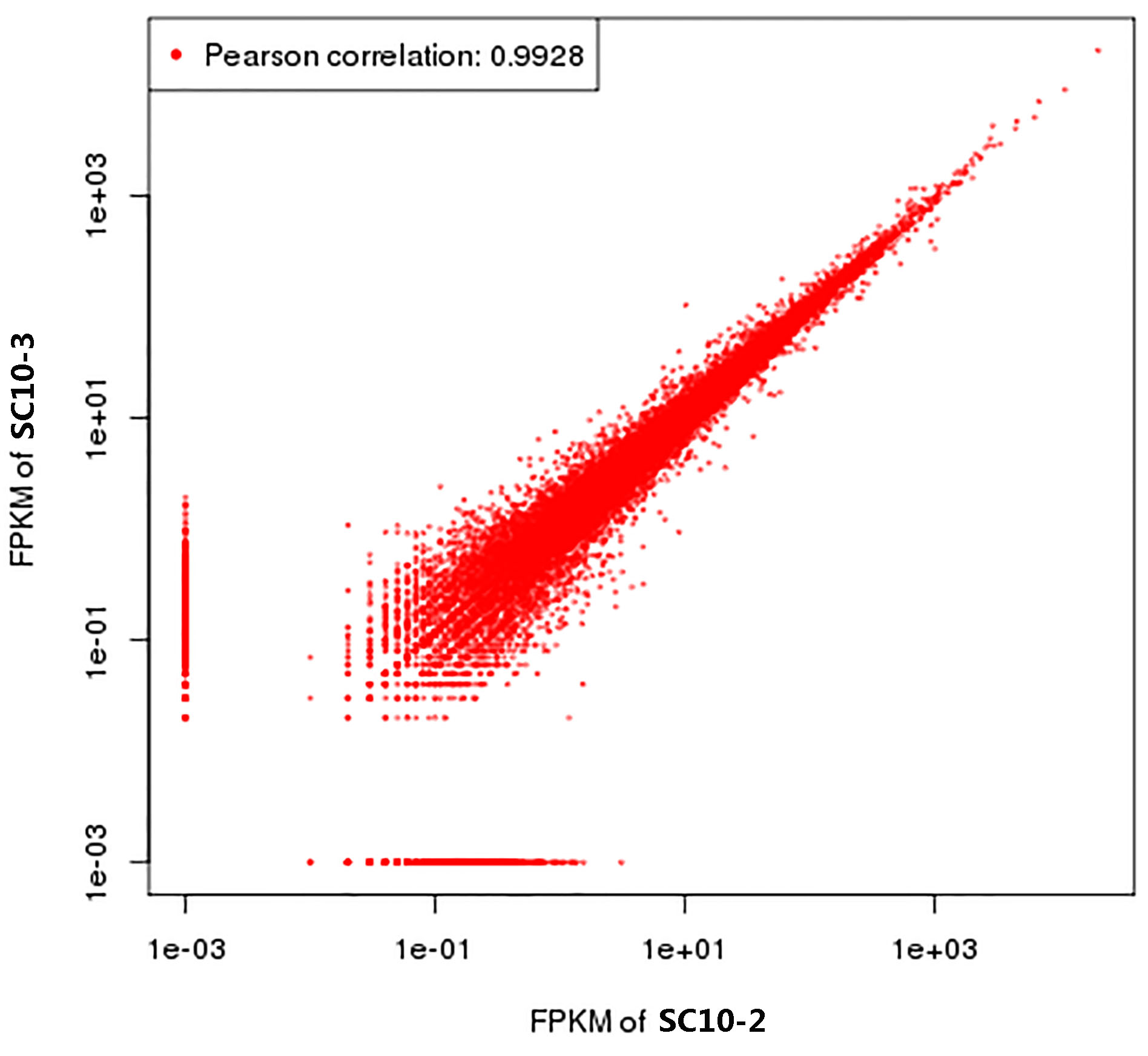

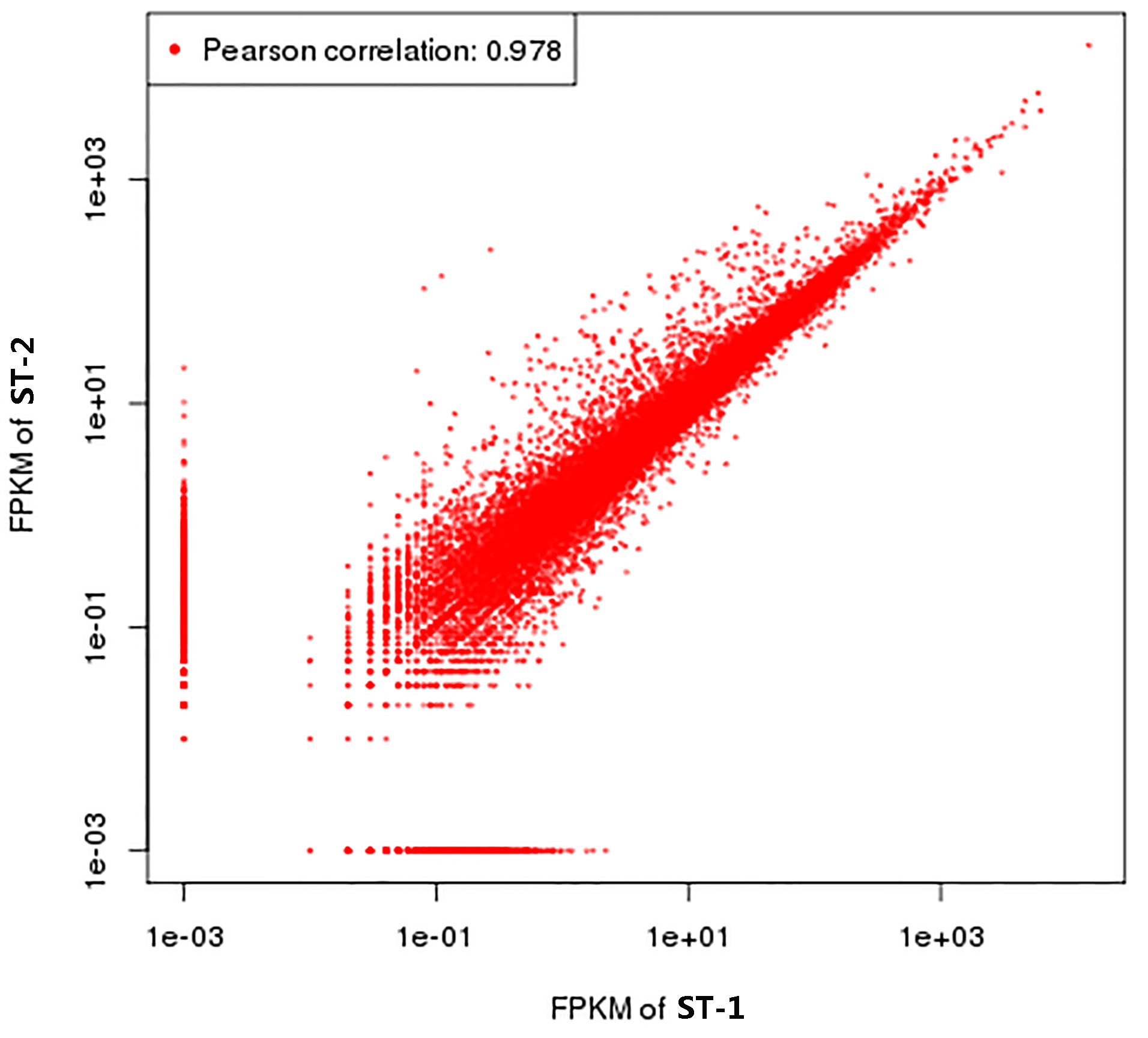

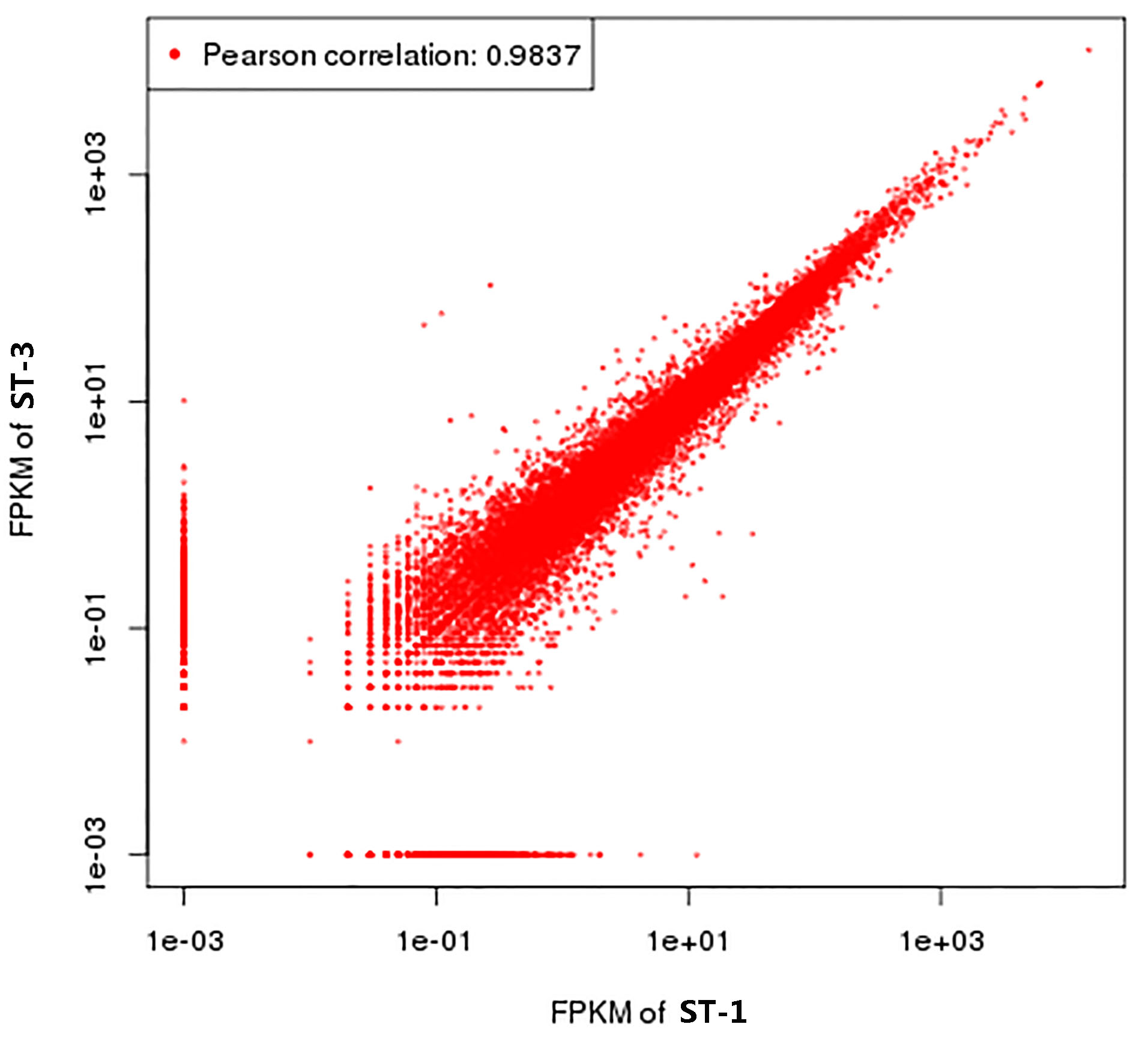


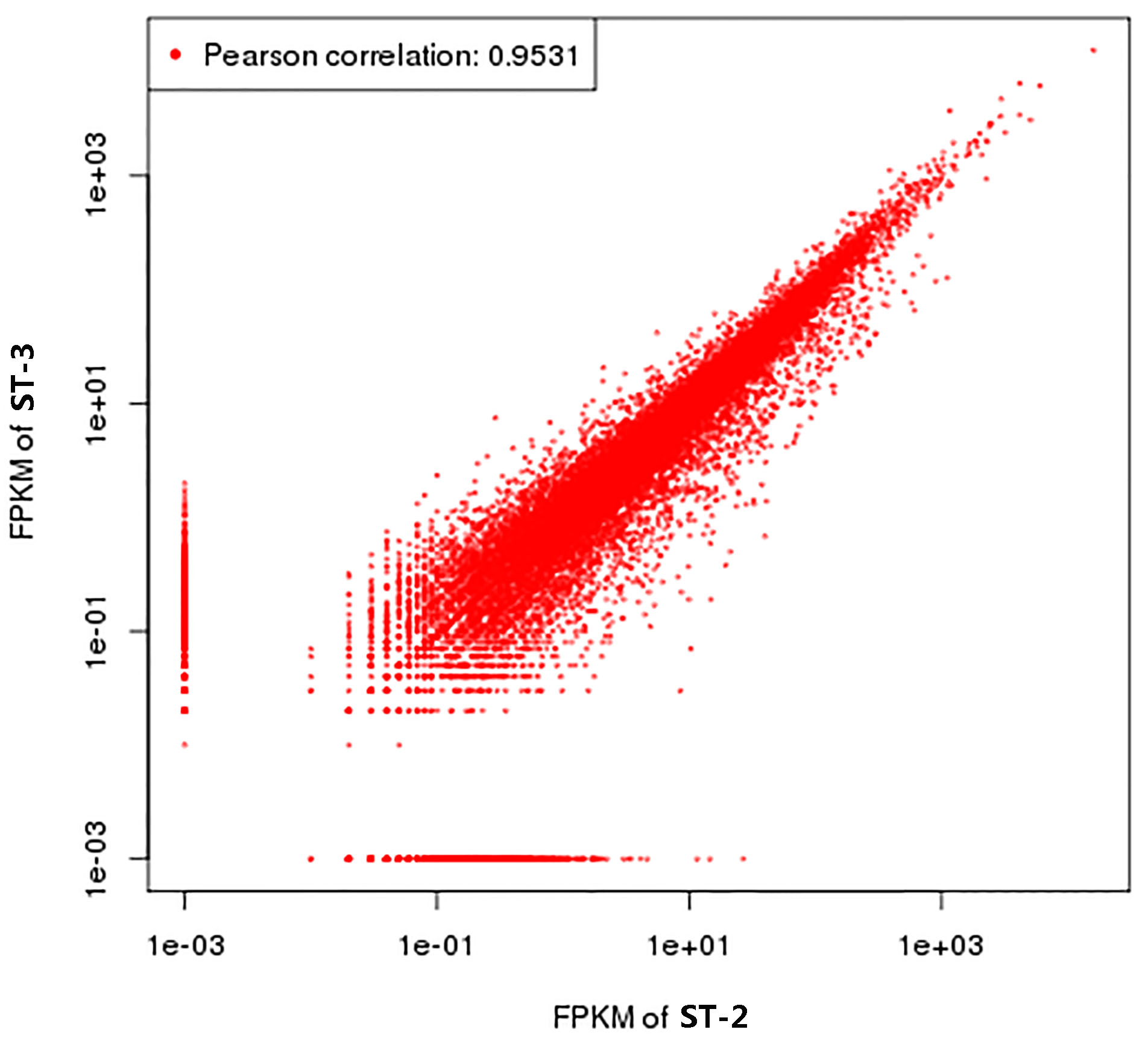


**Figure S2** Reproducibility assay between three parallel experiments of the same variety
